# *Tamarix articulata* Extracts Exhibit Antioxidant Activity and Offer Protection against Hydrogen Peroxide-Mediated Toxicity to Human Skin Fibroblasts

**DOI:** 10.1155/2020/8896263

**Published:** 2020-11-19

**Authors:** Abdullah M. Alnuqaydan

**Affiliations:** Department of Medical Biotechnology, College of Applied Medical Sciences, Qassim University, Buraydah, Saudi Arabia

## Abstract

*Tamarix articulata* (TA) is a wild halophytic plant growing in extremely harsh environmental conditions in the deserts of Saudi Arabia. Evaluating the protective effect of the methanolic extract of different parts (fresh and dry leaves, stem, and root) of TA was determined by MTT assay using Hs27 skin fibroblasts as the cellular model. The study was designed and conducted in two sets. The first set assesses the toxicity profile of TA extracts in both concentration- and time-dependent ways on Hs27 cells. Our MTT results showed that methanolic extracts from all four parts of TA at varying doses (27.5, 55, 110, and 220 *μ*g/mL) display negligible toxicity when exposed for 4 h. However, exposure of Hs27 cells to varying doses of all four TA extracts for 24 and 48 h promotes significant 23%, 24%, 26%, and 25% (*p* < 0.05) and 35%, 36%, 39%, and 41% (*p* < 0.05) cell toxicity at 220 *μ*g/mL of all four TA extracts compared to untreated control cells. To evaluate the protection offered by TA extracts against H₂O₂, we perform a second set of experiments to preincubate Hs27 cells with the TA extracts in both dose- and time-dependent way. This is followed by 300 *μ*M hydrogen peroxide- (H₂O₂-) mediated oxidative insult for 1 h. Using MTT assay, we found that methanolic extracts of TA at different time points (4, 24, and 48 h) and higher doses (220 *μ*g/mL) provide significant protection in cell viability when challenged with H_2_O_2_-induced oxidative stress in Hs27 cells. The protective effect was more pronounced at 48 h and 220 *μ*g/mL and the amounts were 39%, 41%, 41%, and 44% for stem, root, fresh leaf, and dry leaf TA extracts (*p* < 0.05), respectively, compared to untreated cells (2–4%). Collectively, the current study demonstrates that methanolic extracts of TA contain potential bioactive compounds and offer significant protection against H_2_O_2_-mediated oxidative stress in Hs27 skin fibroblasts.

## 1. Introduction

Polyphenolic bioactive compounds derived from plants are of great importance in the medical sciences because of their therapeutic potential as chronic disease protectors and chemopreventive agents [1]. Owing to their chelating and antioxidant activities, polyphenolic bioactive compounds act as free radical scavengers and neutralize dangerous reactive oxygen species (ROS) [2]. Previous reports in the field of free radical biology revealed that free radicals play a crucial role in the pathophysiology of many chronic diseases including cancer [3]. High level of free radical oxygen species including singlet oxygen, superoxide anion, and H_2_O_2_ promotes oxidative stress cells. These free radicals are unstable and extremely harmful for the biological system; they interact with cellular components and cause damage to cell membrane proteins, lipids, and DNA molecules [4]. The antioxidants within the cells function by firstly scavenging and neutralizing the production of free radicals to prevent any damage and secondly making inroads to disrupt potentially destructive reactions [5].

The major sources of antioxidants are plants and their derived products such as fruits [6]. Plant and herbal products have been studied previously to evaluate their antioxidant potential against oxidative stress [7]. Currently, plant-derived products that are abundant in antioxidants are receiving more attention because they offer numerous health benefits [8]. These plant-derived products contain phenolic compounds which display antioxidant properties [9]. Owing to many phenolic hydroxyl groups, these compounds can scavenge free radicals through the phenoxide ion delocalization process [10].

*Tamarix articulata* (TA) which is commonly called Athal in the Arabic language is a halophytic plant [11]. Owing to its fine interaction with salinity regulated microbial communities to maintain optimum salt concentration around the roots, the plant can grow in extremely harsh conditions [12]. The plant belongs to the Tamaricaceae family and may reach a height of 20 meters [13]. From ancient times, TA has been used as a fork medicine by the Tafilalet population—a tribal people in the south-eastern region of Morocco—against various ailments including hair loss, hypertension, ulcers, and gastrointestinal disturbances [14, 15]. Previous findings revealed the presence of numerous bioactive compounds in TA extract exhibit curative effects against epilepsy [15]. The major phytochemical constituents of TA extract which are responsible for pharmacological activities of TA extract are presented in Table 1 [21]. Additionally, dry leaves of TA have been used to cure skin diseases in Saudi Arabia [22]. Preliminary studies have revealed that the extract of TA from the Moroccan region reveals few biological activities [23]. Therefore, we investigate whether TA extract could also have the potential to protect cells against H₂O₂-mediated oxidative stress. Using cell cytotoxicity (MTT), the current study evaluates the cytotoxicity profile and antioxidant potential of methanolic extracts of TA after being challenged with hydrogen peroxide- (H₂O₂-) mediated oxidative stress in Hs27 skin fibroblasts.

## 2. Materials and Methods

### 2.1. Collection of Plant Material and Preparation of Extracts

The plant material (TA) was collected in August 2019 from the desert regions of Qassim province of the Kingdom of Saudi Arabia.

Extracts of all parts of TA were formulated by the standard protocol mentioned in our previous article [24, 25]. All the parts (fresh leaves, dry leaves collected from the floor, stem, and root) were first air-dried in the shade to remove moisture completely. After being chopped into small pieces, all parts of TA were ground in a kitchen blender to produce a fine powder. After weighing 12 g of powder of each part of TA, the power was added to 300 mL of 100% methanol and stirred constantly for 5 days at room temperature [24]. The mixture obtained after stirring was filtered through a cheesecloth to remove the bulk followed by filtering through a Whatman filter paper in an autoclaved glass beaker. Then the methanol (solvent) was completely evaporated from the plant extract mixture in a glass beaker by keeping the temperature of the hot plate at 45°C. This was done to avoid degrading the heat labile compounds. Following this, the fine powder of residue was left in the glass beaker after the solvent's evaporation and stored at 4°C for future experiments to evaluate the biological activities of the TA plant.

### 2.2. Chemicals

The chemicals 1, 1,-diphenyl-2-picryl-hydroxyl (DPPH, #D9132), dimethyl sulphoxide (DMSO, #D8418), 3-(4, 5-dimethylthiazol-2-yl)-2, 5-diphenyltetrazolium bromide (MTT, #M5655), Folin–Ciocalteu reagent (FCR, #F9252), methanol (#646377), fetal bovine serum (FBS, #F0926), phosphate-buffered saline (PBS, #3818), quercetin (QE, #Q4951), and hydrogen peroxide (H₂O₂, #H1009) were procured from Sigma Aldrich.

### 2.3. Cell Culture and Treatments

Hs27 (human skin fibroblast) cell line was ordered and purchased from the American Type Culture Collection (ATCC). The cell line was cultured at Roswell Park Memorial Institute (RPMI)-1640 culture media added with 10% fetal bovine serum (FBS) and 1% penicillin-streptomycin solution under an aseptic condition in 5% CO_2_ humidified incubator. The Hs27 cell line was regularly checked for Mycoplasma contamination.

### 2.4. Determination of Total Polyphenols

The determination of total phenols of TA extracts (methanolic) was determined by the standard Folin–Ciocalteu reagent (FCR) method. The standard compound gallic acid (GA) was taken as a reference compound to determine total phenols in the TA extract [26]. Briefly, 1 ml each of FCR and extract (1 mg/mL) of various parts of TA in a glass tube were mixed properly using a vortex for 5 minutes. After proper mixing of the 3 ml substance, sodium carbonate (2%) was added, and the mixture was properly mixed again for 5 min to ensure this was thorough. The mixture in glass tubes was allowed to incubate in the dark for 3 h at room temperature. By using a spectrophotometer, the absorbance at 760 nm of each solution in the glass tubes was determined against the blank which contains all reagents except test samples or gallic acid under similar conditions. All the readings/absorbance were carried out in triplicate. The total polyphenolic content is expressed as milligrams of GA equivalent per gram dry weight (mg/GAE/g DW). The following formula was used to calculate the phenolic content:

(1) $$\begin{array}{c} C=\frac{c\times V}{m}. \end{array}$$

Here, *C* is total phenolic content expressed in mg GAE/g dry weight, *c* is concentration of gallic acid in mg/mL obtained from the calibration curve, *V* is volume of extract in mL used, and *m* is mass of extract in g used.

### 2.5. Determination of Total Antioxidant Capacity (TAC)

The TAC of TA extracts was determined by a standard method [27] with only slight modifications. Briefly, 0.5 mL of varying concentrations of TA extracts along with standard compound (ascorbic acid) was mixed with a 3 mL mixture (1% ammonium molybdate, 28 mM sodium phosphate, and 0.6 M sulphuric acid) in the test tubes. After ensuring complete mixing by gentle vortex, the mixtures in the glass test tubes were incubated for 10 min at 95°C. Once the reaction was completed, the reaction mixture was cooled down at room temperature and using a spectrophotometer, optical density of the reaction mixture was measured at 695 nm after being normalized with a blank. Increase in optical density of the reaction mixture is directly proportional to the TAC of TA extracts.

### 2.6. Hydroxyl Radical Scavenging Activity (HRSA)

The HRSA activity of TA extract was determined by standard method [28] with some modification. Briefly, 0.5 ml of varying concentrations of TA extract along with standard was added into a glass test tube. Each test tube was added with 1 mL (0.85% DMSO), 0.5 mL (0.018% EDTA), 0.5 mL (22% ascorbic acid), and 1 mL (0.13% iron + 0.26% EDTA solution). After adding these reagents and then capping tightly, the test tubes were incubated for 15 min at 85°C. Immediately after incubation, the test tubes mL had 0.5 mL 17.5% precooled TCA added to them, followed by the addition of 3 mL of Nash reagent (300 *μ*L glacial acetic acid, 7.5 g ammonium acetate, and 200 *μ*L acetyl acetone mixed in 100 mL distilled water) in each tube. They were then incubated for the next 15 min at room temperature. After incubation, the optical density of each solution in the test tubes was measured at 412 nm of wavelength utilizing a spectrophotometer. The percentage of HRSA was calculated by applying the following equation:

(2) $$\begin{array}{c} \text{\% HRSA}=\frac{\text{absorbance of control }\left(A_{\text{o}}\right)-\text{absorbance of TA extract}}{\text{standard compound }\left(A_{1}\right)/\text{absorbance of control}\left(A_{\text{o}}\right)}\times 100\text{.} \end{array}$$

The IC_50_ was calculated after calculating percentage of HRSA of varying concentrations of TA extract.

### 2.7. Cell Proliferation/Viability Assay

Cell viability was determined by a well-known MTT assay as per the standard protocol [29]. Briefly, 5 × 10^3^ Hs27 cells were harvested and plated in each well of the 96-well plate and subsequently attached to the bottom of the well overnight at 37°C in 5% CO_2_ humidified incubator. After they had properly adhered to the bottom surface of the well, the cells were exposed to the varying (27.5 *μ*g/mL to 220 *μ*g/mL) doses of each extract along with untreated control for 24 h in 5% CO_2_ humidified incubator. Subsequently, after 24 h, each well-containing cell had 20 *μ*l of MTT solution (2.5 mg/mL) added to it and incubated for 3 to 4 h at 37°C in 5% CO_2_ humidified incubator. The formazan crystals formed by the interaction of MTT dye with succinyl dehydrogenase of mitochondria of live cells were dissolved in DMSO. The solution thus obtained appears to be measured at 570 nm wavelength using a multiplate reader. The absorbance recorded was analyzed and results were expressed as percentage cell viability of TA extract-treated cells compared to the untreated control cells.

### 2.8. Statistical Analysis

To obtain the data from all the experiments, the process was done more than three times. The data analysis of all the independent experiments was performed and analyzed utilizing one-way ANOVA. The data were expressed as the mean of ±SE. The *p* value equal to or less than 0.05 is said to be significant.

## 3. Results and Discussion

### 3.1. Characteristics of TA Extract

Using absolute methanol as solvent for all four parts of TA (dry and fresh leaves, stem, and root) extraction by constant stirring for 5 days, the amount (g/100 g) of yield extracted from all four parts was the highest in dry leaves (12.78%) followed by fresh leaves (9.87%), root (9.13%), and stem (8.34%) as shown in Table 2. The determination of polyphenol content was analyzed by Folin–Ciocalteu assay which was expressed as gallic acid equivalent (GAE) per gram of extract. As shown in Table 2, the polyphenol content for all four extracts of TA is 409.92 mg GAE/g for dry leaf extract followed by 387.08 mg GAE/g for root extract, 141.75 mg GAE/g for stem extract, and 137.12 mg GAE/g for fresh leaf extract. These figures suggest that TA extracts have an abundant quantity of polyphenols and are believed to possess bioactive compounds with potential antioxidant activity.

**Figure 1:**
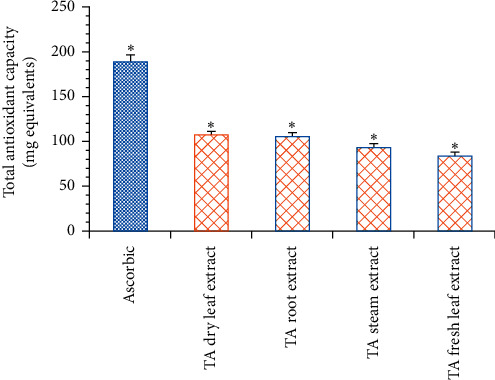
Total antioxidant capacity of all four parts (dry leaf, root, stem, and fresh leaf methanolic extract) of TA plant compared with standard compound ascorbic acid expressed in mg equivalents. Data represented as the mean ± SEM; *n* = 3 and ^*∗*^*p* < 0.05.

Our group's previous study revealed that owing to the presence of an abundant quantity of polyphenolic compounds in all four parts of TA extract and related species, they exhibited promising antioxidant activity by DPPH assay [25, 30]. To support and validate the antioxidant potential of TA extracts, we evaluated the total antioxidant capacity (TAC) of all four TA extracts via phosphomolybdate assay. Our results demonstrated that the highest antioxidant capacity was shown by standard compound ascorbic acid (187.23 ± 10.34 mg equivalents) followed by dry leaf extract (107.21 ± 5.38 mg equivalents), root extract (105.72 ± 6.19 mg equivalents), stem extract (92.77 ± 4.17 mg equivalents), and fresh leaf extract (83.27 ± 5.31 mg equivalents) (Figure 1 and Table 3). Additionally, our hydroxyl radical scavenging activity suggests that all four parts of TA extracts showed dose-dependent antioxidant activity with IC_50_ value 50.19 ± 3.34 *μ*g/mL, 51.33 ± 3.51 *μ*g/mL, 59 ± 3.67 *μ*g/mL, and 81.34 ± 5.34 *μ*g/mL for dry leaf, root, stem, and fresh leaf extract compared with standard compound ascorbic acid which has a IC_50_ value of 5.9 ± 0.31 *μ*g/mL (Table 3). Together, these results suggest that all four parts of TA extract exhibit promising antioxidant activity that is able to neutralize reactive oxygen species. In this way, protection against free radicals produced during metabolic reactions is offered.

### 3.2. *In Vitro* Toxicity Profile of TA Extracts on Human Skin Fibroblast (Hs27) Cells

Numerous reports suggest that the evaluation of cellular toxicity and protective effect of plant extracts is necessary to document whether the extract is safe to use against various harmful effects caused by free radicals [31]. Under physiological conditions, the free radicals and ROS are generated from aerobic metabolism [32]. If not efficiently neutralized by our antioxidant mechanism in our body, these oxidants are very reactive. They quickly interact with membrane proteins as well as intracellular components such as DNA molecules and cause damage which leads to a pathological state [33]. Free radicals and ROS are associated with numerous diseases which include neurodegenerative disease, cancer, diabetes, inflammation, and atherosclerosis [34]. Polyphenolic natural compounds have been documented as possessing antioxidant activity and have the potential to scavenge free radicals and ROS [35]. Plants are abundant sources of antioxidant polyphenols and other compounds such as tocopherol, phycocyanin, and essential fatty acids [36]. Natural compounds derived from plants can be used in remedies found in cosmetic products to enrich the antioxidant content of skin cells against free radicals and ROS generated by environmental toxicants [37].

**Figure 2:**
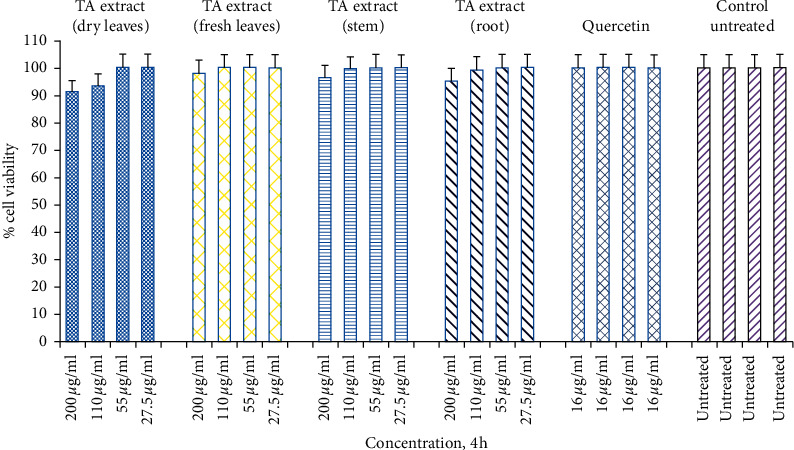
Cell viability determined by MTT assay. Hs27 skin fibroblasts were seeded at a density of 10,000 cells/well in 96-well plates and allowed to incubate overnight to attach to the surface. Next day, treat the cells with TA extracts (fresh and dry leaf, stem, and root extracts) for 4 h along with quercetin as a positive control (16 *μ*g/mL) and control as untreated. Data shown as mean ± SEM; *n* = 3 and ^*∗*^*p* < 0.05.

**Figure 3:**
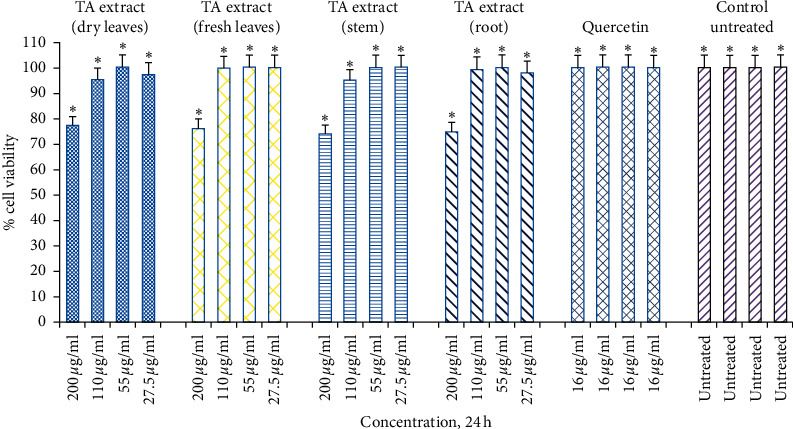
Cell viability determined by MTT assay. Hs27 skin fibroblasts were seeded at a density of 10,000 cells/well in 96-well plates and allowed to incubate overnight to attach to the surface. Next day, treat the cells with TA extracts (fresh and dry leaf, stem, and root extracts) for 24 h along with quercetin as a positive control (16 *μ*g/mL) and control as untreated. Data shown as mean ± SEM; *n* = 3 and ^*∗*^*p* < 0.05.

**Figure 4:**
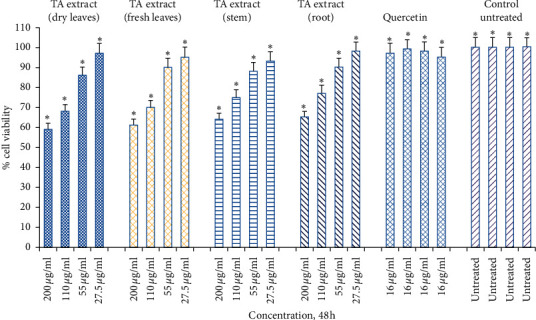
Cell viability determined by MTT assay. Hs27 skin fibroblasts were seeded at a density of 10,000 cells/well in 96-well plates and allowed to incubate overnight to attach to the surface. Next day, treat the cells with TA extracts (fresh and dry leaf, stem, and root extracts) for 48 h along with quercetin as a positive control (16 *μ*g/mL) and control as untreated. Data shown as mean ± SEM; *n* = 3 and ^*∗*^*p* < 0.05.

A few years ago, Park et al. [38] demonstrated the importance of evaluating the toxicity profile of plant extract (*Cordyceps militaris* extract) using skin fibroblasts as a cellular model [38]. The study further reveals that exposure of dermal skin fibroblasts to 0.8 mM dose of H_2_O_2_ for 3 h reduces cell viability by 59% when compared with untreated control skin fibroblasts. However, pretreatment of dermal skin fibroblasts with *Cordyceps militaris* extract in a dose-dependent way (50 to 100 *μ*g/mL) reduces the cell death of dermal skin fibroblasts significantly, thus preventing H_2_O_2_-mediated oxidative stress-mediated cell death. Consistent with these findings, the current study sought to investigate the *in vitro* toxicity and protection offered by TA extracts on a human skin fibroblast (Hs27). The experimental setup was designed for both dose- (27.5, 55, 110, and 220 *μ*g/mL) as well as time-dependent (4, 24, and 48 h) manner. We observed a significantly negligible toxicity at both lower and higher doses (5–9%, ^*∗*^*p* < 0.05) of TA extracts when Hs27 cells were exposed for 4 h in comparison to the untreated control (Figure 2). Similarly, when Hs27 fibroblasts were exposed for 24 h, the toxicity of cells at lower doses was insignificant (1–5%, *p* < 0.05), whereas, at the highest dose (220 *μ*g/mL), we found significant toxicity in the 23–26% range, *p* < 0.05 (Figure 3). However, for 48 h time point, skin fibroblasts exposed to varying doses of TA extracts revealed that significant toxicity (17–22%, 35–41%; *p* < 0.05) was observed at higher doses (110, 220 *μ*g/mL), respectively (Figure 4).

These results indicate that TA extracts at smaller doses have a small amount of bioactive substances and probably are diffused across the plasma membrane in low concentrations to impart any toxicity effect. Therefore, the highest doses of plant extract which are beyond the concentrations used in commercial products would have bioactive constituents in adequate quantities to be toxic. Intriguingly, the outcome of this effect depends on the duration of interaction between the extract components and the cells. Thus, this interaction will decide the uptake of bioactive components by cells and their impact on cell viability. Collectively, these results demonstrate that the lower doses of such plant extracts, firstly, display a safe toxicity profile and, secondly, are not deleterious to skin fibroblasts. Therefore, being incorporated into the commercial products in smaller doses will enhance their effectiveness.

The toxicity of cells upon being treated with larger doses of TA extract and longer exposure could be due to the presence of bioactive compounds present in the extract. Alternatively, the decrease in cell viability at higher doses could be due to the presence of some prooxidant activity, and extortion of conditional media for a longer period of time (48 h) could increase the toxicity on skin fibroblasts; however, at lower doses, the prooxidant effect is negligible. This explanation is well supported by reports that the TA extracts exert a significant antiproliferative effect against a panel of human tumor cell lines at both dose- and time-dependent contexts [25].

### 3.3. TA Extracts Induce Protection against H₂O₂-Mediated Oxidative Stress in Human Skin Fibroblasts

The generation of free radicals and ROS leads to oxidative stress which in turn leads to various ailments in the human body [39]. H_2_O_2_ is one of the most common forms of ROS, generated in the cellular models when exposed to UV radiation and pollutants. Depending on the level of H_2_O_2_ in the cellular system, it may help in wound healing by acting as a secondary messenger and remodulating signaling to repair damaged tissue. Nonetheless, excessive production of H_2_O_2_ may lead to inflammation and induce oxidative stress in cellular models [40]. Excessive production of H_2_O_2_ promotes oxidative damage to cellular models by augmenting MAPK-dependent cell death mechanisms [41]. Previous reports suggest that a significant increase in the H₂O₂ level and concomitant decrease in catalase activity was observed in dermal fibroblasts of aged human skin than the dermal fibroblasts of younger human skin [42].

The vulnerable targets of free radicals and ROS are cell membrane, lipids, DNA, and proteins [43]. Under oxidative stress conditions, the body's own antioxidant system is not sufficient to neutralize free radicals and ROS effectively; hence, it is dependent on exogenous sources [44]. Owing to the presence of high antioxidant compounds, the plants are the main exogenous source of antioxidants in the form of fruits, dietary products, and cosmetic products derived from natural sources to enrich the antioxidant level in our body to neutralize free radicals and ROS effectively [45, 46]. Various plant extracts can display antioxidant potential by neutralizing free radicals produced during physiological reactions [47]. Therefore, we exposed Hs27 cells to varying concentrations (27.5, 55, 110, and 220 *μ*g/mL) of TA extract for different time points (4, 24, and 48 h), followed by 300 *μ*M of H₂O₂ exposure for 1 h [48, 49], to evaluate the protective effect of TA extract. The cell killing induced by H₂O₂-mediated by oxidative stress in untreated cells was significantly different from cells pretreated with TA extract in both dose- and time-dependent ways. Owing to the production of ROS, the percentage of cell viability varies when H₂O₂-mediated oxidative stress was induced. Pretreatment with TA extracts offers significant protection at higher concentrations as well as different time points in skin fibroblasts against H₂O₂-mediated cell death.

**Figure 5:**
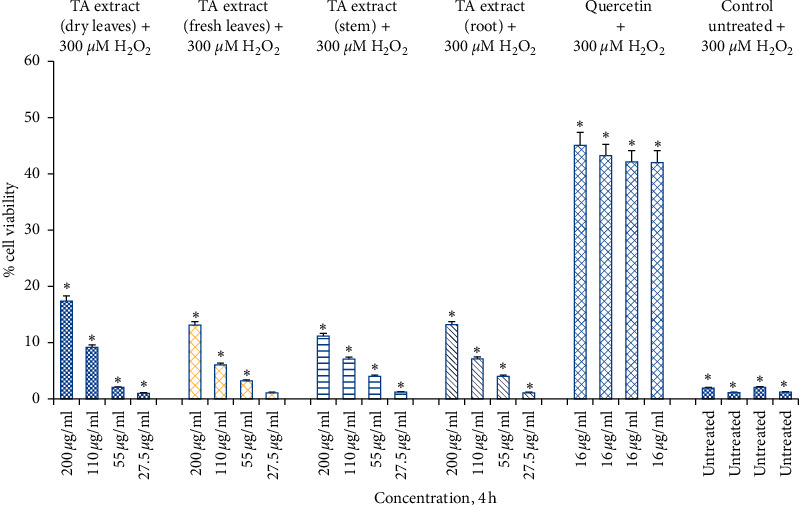
Cell viability determined by MTT assay. Hs27 skin fibroblasts were seeded at a density of 10,000 cells/well in 96-well plates and allowed to incubate overnight to attach to the surface. Next day, treat the cells with TA extracts (fresh and dry leaf, stem, and root extracts) for 4 h along with quercetin as a positive control (16 *μ*g/mL) and control as untreated. After the completion of treatment, expose the pretreated cells with 300 *μ*M of H₂O₂ for 1 h. Data shown as mean ± SEM; *n* = 3, ^*∗*^*p* < 0.05.

Previous studies revealed that quercetin reduces the oxidative damage of skin cells by acting as a powerful protectant against H₂O₂-mediated oxidative insults and effectively protects cell death mechanisms at a 50 *μ*M dose [50]. Therefore, we use quercetin as the positive control in our study to evaluate the level of protection against H₂O₂-mediated oxidative stress. In comparison to TA extracts, the positive control quercetin (16 *μ*g/mL) protects cells significantly at all time points when exposed to H₂O₂-mediated oxidative stress for 1 h. Pretreatment with varying doses (27.5, 55, 110, and 220 *μ*g/mL) of TA extract for 4 h, followed by H₂O₂-mediated oxidative insult, offers protection in a dose-dependent manner. Significant protection (11–17%) was offered at 220 *μ*g/mL, followed by 6–9% at 110 *μ*g/mL; however, positive control quercetin offers 42–45% protection against H₂O₂-mediated oxidative stress (Figure 5).

**Figure 6:**
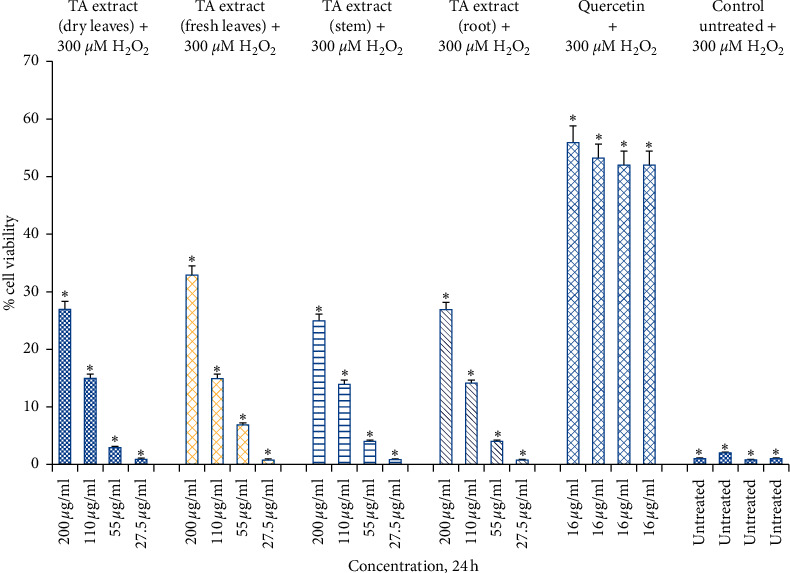
Cell viability determined by MTT assay. Hs27 skin fibroblasts were seeded at a density of 10,000 cells/well in 96-well plates and allowed to incubate overnight to attach to the surface. Next day, treat the cells with TA extracts (fresh and dry leaf, stem, and root extracts) for 24 h along with quercetin as positive control (16 *μ*g/mL) and control as untreated. After the completion of treatment, expose the pretreated cells with 300 *μ*M of H₂O₂ for 1 h. Data shown as mean ± SEM; *n* = 3, ^*∗*^*p* < 0.05.

**Figure 7:**
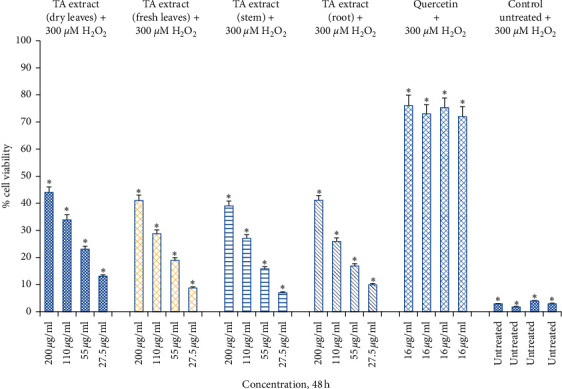
Cell viability determined by MTT assay. Hs27 skin fibroblasts were seeded at a density of 10,000 cells/well in 96-well plates and allowed to incubate overnight to attach to the surface. Next day, treat the cells with TA extracts (fresh and dry leaf, stem, and root extracts) for 48 h along with quercetin as a positive control (16 *μ*g/mL) and control as untreated. After the completion of treatment, expose the pretreated cells with 300 *μ*M of H₂O₂ for 1 h. Data shown as mean ± SEM; *n* = 3, ^*∗*^*p* < 0.05.

As evidenced by the high survival percentage of Hs27 fibroblasts in solely TA extract treatment compared to pretreatment with TA, this was followed by H₂O₂-mediated oxidative stress (Figures 2 and 5). Similarly, significant protection at 14–15% and 25–33% at doses of 110 *μ*g/mL and 220 *μ*g/mL, respectively, were noticed when Hs27 fibroblasts were pretreated with TA extract for 24 h followed by oxidative stress with H₂O₂ for 1 h (Figure 6). Although we observe a maximum cell toxicity effect after 48 h of treatment with larger doses of TA extract alone (Figure 4), pretreatment of Hs27 cells with TA extracts in a dose-dependent way for 48 h was followed by 1 h exposure to H₂O₂-mediated oxidative stress. We observed significant protection at 16–23%, 26–34%, and 39–44% at doses of 55 *μ*g/mL, 110 *μ*g/mL, and 220 *μ*g/mL, respectively (Figure 7). The relative cell survival after pretreatment with TA extracts followed by exposure to H₂O₂ for 1 h was significantly higher than untreated cells following exposure to H₂O₂. The high percentage of protection offered to Hs27 fibroblasts after 48 h exposure to TA extracts followed by oxidative stress-induced by H₂O₂ could be due to enough time being required for bioactive compounds. These are present in TA extract and can enter the cells and offer protection by neutralizing free radicals generated during exposure to H₂O₂. Although the higher concentrations for a longer period of time show significant cell death, at lower doses for a longer time point, they do not exhibit toxic effects to Hs27 fibroblasts. Nonetheless, they permit bioactive compounds to pass through the cell membrane. These bioactive compounds have antioxidant potential and protect cells against any exposure to free radicals generated by H₂O₂.

## 4. Conclusion

The present study demonstrates that all four parts of TA extracts contain large amounts of polyphenolic bioactive compounds and exhibit promising antioxidant potential. Dose- and time-dependent exposure of skin fibroblasts (Hs27 cells) to TA extracts reveals a negligible cytotoxicity to Hs27 cells when exposed for 4 h. However, 24 h and 48 h TA extract treatment to Hs27 cells exhibits significant toxicity. Additionally, we observe that TA extracts offer protection to Hs27 skin fibroblasts against free radicals generated by H₂O₂-mediated oxidative stress. These kinds of extracts can potentially be used in cosmetic products to offer protection to skin fibroblasts against free radicals generated by environmental toxicants and UV radiation. However, further research is very much needed to evaluate the underlying mechanism for the protective effect of TA extracts (dry leaf extract) using human skin fibroblasts as cellular models.

## Figures and Tables

**Table 1:** Major constituents of TA that exhibit pharmacological activity.

S. no.	Major constituents	Functions	References
1	Gallic acid	Inhibits lipid peroxidation, promotes radical scavenging activity, and maintains endogenous defense system	[16]
2	Rutin	Exhibits various pharmacological activities such as antioxidant, antidiabetic, anticonvulsant, and neuroprotection activity.	[17]
3	Naringin	Exhibits promising antioxidant, antiproliferative, immunomodulatory, and immunostimulatory potential in cellular models	[18]
4	Quercetin	Promising antioxidant and antiproliferative activity, inhibitory effect against acetyl cholinesterase enzymes	[19]
5	Chlorogenic acid	Exhibits free radical scavenging activity, hepatoprotective activity, anti-inflammatory, and immune modulation activity against various cellular models	[20]

**Table 2:** Type of extraction used for extraction from medicinal plant TA and its yield (g/100 g of DW of powder) and total polyphenolic content of different parts of TA.

S. no.	Medicinal plant organs	Extraction by stirring in methanol	Polyphenolic content (mg GAE/g DW)
1	TA dry leaves	12.7%^*∗*^	409.92 ± 6.03^*∗∗*^
2	TA fresh leaves	9.87%^*∗*^	137.12 ± 5.01^*∗∗*^
3	TA stem	8.34%^*∗*^	141.75 ± 4.21^*∗∗*^
4	TA root	9.13%^*∗*^	387.08 ± 5.93^*∗∗*^

Data shown as mean ± SEM; *n* = 3, ^*∗*^*p* < 0.05, and ^*∗∗*^*p* < 0.01.

**Table 3:** Total antioxidant capacity and hydroxyl radical scavenging activity of different parts of TA along with standard compound ascorbic acid.

S. no.	Name of samples	Total antioxidant capacity (mg equivalents)	Hydroxyl radical scavenging activity IC₅₀ values (*μ*g/mL)
1	Ascorbic acid	187.23 ± 10.34^*∗*^	5.9 ± 0.31^*∗*^
2	TA dry leaf extract	107.21 ± 5.38^*∗*^	50.19 ± 3.34^*∗*^
3	TA root extract	105.72 ± 6.19^*∗*^	51.33 ± 3.51^*∗*^
4	TA stem extract	92.77 ± 4.17^*∗*^	59 ± 3.67^*∗*^
5	TA fresh leaf extract	83.27 ± 5.31^*∗*^	81.34 ± 5.34^*∗*^

Data shown as mean ± SEM; *n* = 3 and ^*∗*^*p* < 0.05.

## Data Availability

The data used to support the study are available upon request to the corresponding author.

## Abbreviations

ROS: Reactive oxygen species
H_2_O_2_: Hydrogen peroxide
FCR: Folin–Ciocalteu reagent
DPPH: 1,1-Diphenyl-2-picryl-hydroxyl
DMSO: Dimethyl sulphoxide
MTT: 3-(4,5-Dimethylthiazol-2-yl)-2,5-diphenyltetrazolium bromide
FBS: Fetal bovine serum
PBS: Phosphate-buffered saline
QE: Quercetin
GA: Gallic acid
SE: Standard error
ANOVA: Analysis of variance
Na_2_CO_3_: Gallic acid
